# Benign Calcified Axillary Leiomyoma in a Young Male: A Case Report

**DOI:** 10.7759/cureus.85059

**Published:** 2025-05-29

**Authors:** Jose A Parra Ayala, Sunny Rampuria, Sabrine Semoin

**Affiliations:** 1 General Surgery, St. George's University School of Medicine, St. George's, GRD; 2 General Surgery and Dermatology, Ross University School of Medicine, Bridgetown, BRB; 3 General Surgery, Jackson Health System, Miami, USA

**Keywords:** axillary leiomyoma, benign, dystrophic calcification, extrauterine, leiomyosarcoma, malignant

## Abstract

Leiomyomas are benign smooth muscle tumors commonly originating from the uterus and gastrointestinal tract. Their appearance in extrauterine locations, such as the axilla, is rare, presenting a diagnostic challenge. Within this report, we highlight a case of a longstanding leiomyoma within the right axilla, associated imaging, and histopathological findings, as well as the prognosis of a 30-year-old male patient.

## Introduction

Leiomyomas are benign neoplasms originating from the hypertrophy of smooth muscle cells, predominantly in the uterus and gastrointestinal tract. Leiomyomas within the uterus tend to manifest symptoms of abdominal pain, constipation, pelvic pressure, possible reproductive dysfunction, abnormal uterine bleeding, menstrual cycle irregularities, and rare transformation into malignant leiomyosarcomas [1]. Contrasted with the gastrointestinal tract, leiomyomas in this location are prone to producing bleeding and digestive disorders [2].

These masses are typically diagnosed using an ultrasonography; however, magnetic resonance and computer tomography might also be useful [3]. Ultrasound is widely used as the initial imaging modality for detecting uterine leiomyomas. On sonographic examination, leiomyomas typically appear as well-defined, hypoechoic masses that may exhibit heterogeneous echotexture and posterior acoustic shadowing due to calcifications. They often cause distortion of the normal uterine contour. However, ultrasound has limited capability in differentiating benign leiomyomas from malignant uterine sarcomas, especially when sarcomas may present upon imaging with qualities identical to benign leiomyomas [4]. While ultrasound is practical and accessible, its diagnostic accuracy may be insufficient in cases where malignancy must be ruled out.

An MRI offers superior diagnostic performance compared to ultrasound, particularly in identifying uterine leiomyomas. MRIs demonstrate a pooled sensitivity of 0.90 (95% CI: 0.84-0.94), pooled specificity of 0.96 (95% CI: 0.96-0.97), positive likelihood ratio of 13.55 (95% CI: 6.20-29.61), negative likelihood ratio of 0.08 (95% CI: 0.02-0.32), and an area under the receiver operating characteristic (ROC) curve of 0.9759 in differentiating leiomyomas from sarcomas [4]. These values indicate excellent diagnostic accuracy for diagnosing benign leiomyomas using MRI. MRIs allow for a more accurate assessment of tissue characteristics, margins, and internal architecture, making it a more reliable diagnostic tool for evaluating features suggestive of malignancy, such as heterogenous signal intensities, irregular borders, central necrosis, or irregular enhancement [4]. Its diagnostic strength makes MRI the preferred modality in suspicious cases when ultrasound findings are inconclusive. 

In rare situations, these benign neoplasms are produced from extrauterine locations such as the axilla [5]. Leiomyomas are clinically identified in up to 25% of women, with incidence rising during reproductive years, declining after menopause, and rarely occurring in adolescents. While uterine leiomyomas are well-characterized, extrauterine variants remain exceedingly rare and poorly understood, with limited data available on their true incidence. This scarcity of information highlights the need for further research and provides the rationale for presenting this case [6]. 

In this context, we present a case of axillary leiomyoma, highlighting its atypical presentation and the diagnostic challenges associated with such rare occurrences.

## Case presentation

A 30-year-old man presented to the clinic with a deep mass in his right axilla that has been progressively enlarging. The patient mentioned that the mass has been present since childhood and has grown slowly through the years. The mass raised clinical suspicion due to its firm consistency and progressive enlargement without reduction in size. Previous ultrasound-guided needle biopsy of the mass in 2008 showed fibroadipose tissue with calcification. The patient did not report any neurological deficits in the upper extremity or signs of vascular compromise. The patient also denied weight loss and night sweats. The patient claimed that the mass increased in size a few days ago, which prompted his visit.

On physical exam, a 20 x 15 cm indurated, mobile mass with mild tenderness to palpation and well-circumscribed borders was seen. A chest CT revealed a heterogeneous, partially calcified mass in the right axilla measuring approximately 10.5 × 8.6 × 13.6 cm, with no significant enhancement of the soft tissue component. The mass demonstrated interval growth compared to prior measurements, which measured 6.7 × 4.7 cm. In 2008, the mass measured 6.6 × 4.5 cm and was similarly calcified. A probable diagnosis of calcified leiomyoma was made, and an excision of the mass was planned. An ultrasound-guided needle biopsy of the mass prior to surgery showed fragments of stromal tissue with calcific debris and no lymph nodes or evidence of malignancy.

**Figure 1:**
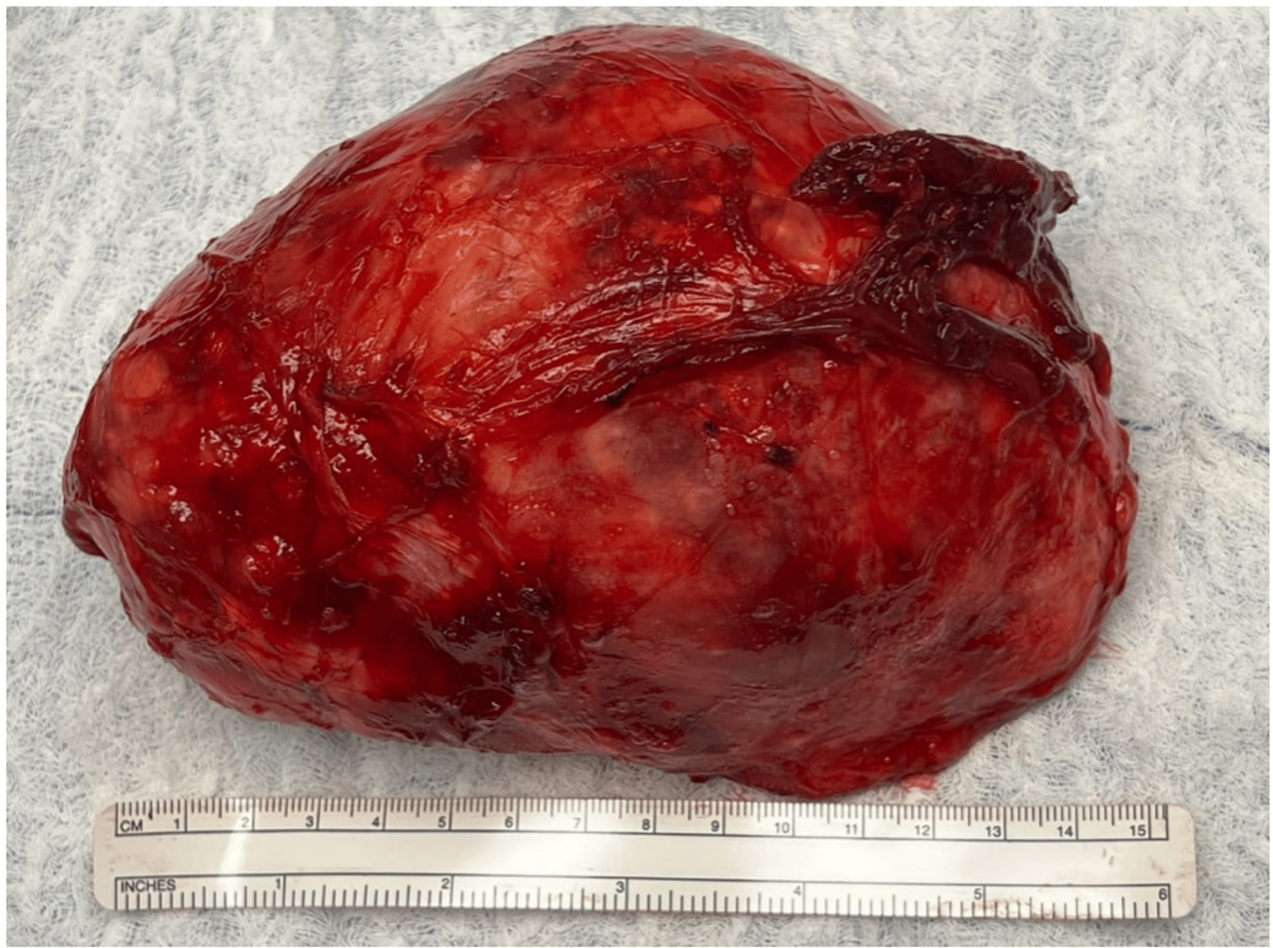
Resected well-defined mass (axillary leiomyoma) from the right axillary region measuring 15 cm in length.

Excision of the specimen was performed successfully, and the patient had an uneventful postoperative course. The excised mass depicted in Figure 1 was sent for pathology.

The final pathology report identified the excised mass as an axillary leiomyoma characterized by extensive dystrophic calcification, focal hyalinization, and infarct-type necrosis. The lesion was well-circumscribed with numerous palisading spindle cells, exhibited no significant cytological atypia, and showed fewer than one mitotic figure per 100 high-power fields. Peripheries of the mass showed a prominent perivascular growth pattern. Immunohistochemistry was positive for smooth muscle actin (SMA), caldesmon, and desmin, and focally positive for cathepsin K. A cluster of cells was positive for MYOD1 and negative for myogenin, S100 protein, SOX10, HMB45, Melan A, CD34, CD117, DOG1, and SS18::SSX. The H3K27me3 expression and a 1% estimated proliferation rate of the Ki67 nuclear labeling index were retained. The presence of MYOD1 expression in leiomyomas is rare, and its significance is unknown. For these collective findings, the pathologist supported the diagnosis of axillary leiomyoma with areas of increased cellularity, which does not warrant worry about malignancy. Surgical resection margins were negative for tumor tissue with a clearance of <0.1 cm.

## Discussion

Leiomyomas are benign tumors often observed in the uterus and digestive system. They can rarely present in different regions of the body, such as the axillary leiomyoma presented in this case [7]. Despite their relatively benign nature, their mass effect can cause symptoms depending on their location and possibly malignant transformation. Proper diagnosis of extrauterine leiomyomas can be challenging on imaging, as incorrect diagnoses can have profound effects on the patient’s management [8]. Diagnosis is difficult to obtain just from imaging. Histopathological examination after surgical excision is required to rule out possible malignancy and obtain a definitive diagnosis [9].

Uterine leiomyomas can be diagnosed on ultrasound, and treatment usually depends on a variety of factors such as the presence of symptoms and hormone alterations [3,10]. Treatments for these masses include selective progesterone receptor modulators and gonadotropin-releasing hormone analogues, hysterectomies for symptomatic uterine fibroids, myomectomies if fertility retention is desired, and uterine artery embolization for more conservative interventions [10]. However, the mainstay and definitive management for extrauterine leiomyomas is a complete surgical excision followed by histopathology to properly diagnose [7,9].

The presented case of a 30-year-old male with a longstanding right axillary calcified mass underscores the diagnostic challenges associated with such atypical presentations.

Clinical presentation and imaging

The patient's initial presentation with a deep, nonspecific axillary mass that remained stable for years before enlarging and becoming calcified is atypical for leiomyomas, which are generally slow-growing. Rapid growth in such tumors raises concerns for potential malignant transformation or alternative diagnoses. A CT scan with IV contrast revealed a heterogeneous, partially calcified mass with minimal enhancement, suggesting a benign etiology. However, axillary masses have a list of differential diagnoses requiring further investigation and imaging to rule out potential malignancies. CT scans and other imaging modalities are essential to differentiate benign calcified masses presenting similarly to the patient in this case from potential malignancies [2]. These differentials include but are not limited to lymphadenopathy, fibroadenomas, hematomas, lipomas, hemangiomas, and carcinomas. Following imaging, a biopsy is recommended to rule out malignancies and provide better advice for the next course of action, such as providing chemotherapeutic regimens for patients with carcinomas. In the case presented above, an excision was recommended for the prevention of potentially malignant transformation of the leiomyoma into a leiomyosarcoma [11].

Histopathological and immunohistochemical findings

Definitive diagnosis was achieved through pathological examination of the resected axillary mass, which showed features characteristic of a leiomyoma. The low proliferation index, indicated by a Ki-67 labeling index of approximately 1%, further supports the benign nature of the tumor [12]. Detailed pathologic examination of the excised mass reveals well-circumscribed margins, dystrophic calcification, focal hyalinization, and low mitotic activity. Immunohistochemical staining was positive for SMA, caldesmon, desmin, and focally positive for cathepsin K, indicating a smooth muscle origin of this mass [13]. Although the findings are suggestive of a benign leiomyoma, malignant transformation, while exceedingly rare, can still occur and should be considered, prompting the decision to proceed with surgical excision [1].

Management and prognosis

Surgical excision is the definitive treatment for symptomatic leiomyomas, regardless of origin, mainly when malignancy cannot be determined preoperatively. In this case, complete resection was achieved with clear margins, and the patient had an unremarkable postoperative recovery period. Due to the benign nature of leiomyomas, patients have a good prognosis with a low probability of recurrence [5].

## Conclusions

This case highlights the importance of including leiomyomas when considering differential diagnoses of axillary calcified masses regardless of location, even in male patients. A complete diagnostic evaluation, including imaging and histopathological assessment, is crucial for accurately diagnosing and adequately managing this condition. Being conscious of such atypical presentations can help clinicians identify and treat comparable cases in the future.
